# Abscisic acid represses the transcription of chloroplast genes*

**DOI:** 10.1093/jxb/ert258

**Published:** 2013-09-28

**Authors:** Maria V. Yamburenko, Yan O. Zubo, Radomíra Vanková, Victor V. Kusnetsov, Olga N. Kulaeva, Thomas Börner

**Affiliations:** ^1^Department of Biology/Genetics, Humboldt University, Chausseestr. 117, D-10115 Berlin, Germany; ^2^Timiriazev Institute of Plant Physiology, Russian Academy of Sciences, Botanicheskaya 35, Moscow 127276, Russia; ^3^Institute of Experimental Botany, AS CR, Rozvojová 263, 165 02 Prague 6, Czech Republic

**Keywords:** Abscisic acid (ABA), chloroplast, cytokinin, *Hordeum vulgare* (L.), nucleus-encoded plastid RNA polymerase (NEP), plastid-encoded plastid RNA polymerase (PEP), photosynthesis, retrograde signalling, regulation of transcription, senescence.

## Abstract

Numerous studies have shown effects of abscisic acid (ABA) on nuclear genes encoding chloroplast-localized proteins. ABA effects on the transcription of chloroplast genes, however, have not been investigated yet thoroughly. This work, therefore, studied the effects of ABA (75 μM) on transcription and steady-state levels of transcripts in chloroplasts of basal and apical segments of primary leaves of barley (*Hordeum vulgare* L.). Basal segments consist of young cells with developing chloroplasts, while apical segments contain the oldest cells with mature chloroplasts. Exogenous ABA reduced the chlorophyll content and caused changes of the endogenous concentrations not only of ABA but also of cytokinins to different extents in the basal and apical segments. It repressed transcription by the chloroplast phage-type and bacteria-type RNA polymerases and lowered transcript levels of most investigated chloroplast genes drastically. ABA did not repress the transcription of *psbD* and a few other genes and even increased *psbD* mRNA levels under certain conditions. The ABA effects on chloroplast transcription were more pronounced in basal vs. apical leaf segments and enhanced by light. Simultaneous application of cytokinin (22 μM 6-benzyladenine) minimized the ABA effects on chloroplast gene expression. These data demonstrate that ABA affects the expression of chloroplast genes differentially and points to a role of ABA in the regulation and coordination of the activities of nuclear and chloroplast genes coding for proteins with functions in photosynthesis.

## Introduction

The plant hormone abscisic acid (ABA) is involved in the control of developmental processes, such as seed and bud dormancy. It suppresses growth and promotes senescence. ABA biosynthesis is stimulated by stress, especially stresses associated with dehydration (drought, salinity, and cold). It plays major roles in the response of plants to these abiotic stress factors and in the defence against pathogens (reviewed in Cao *et al.*, 2011; Qin *et al.*, 2011). ABA receptors and essential components of ABA signalling have been identified (reviewed in Raghavendra *et al.*, 2010). ABA signalling leads to changes in the expression of several thousand nuclear genes and interacts with the signalling networks of other factors such as light (reviewed by Lau and Deng, 2010), sugars (Wingler and Roitsch, 2008) and other hormones (for reviews see Acharya and Assmann, 2009; Qin *et al.*, 2011; Robert-Seilaniantz *et al.*, 2011). The antagonistic actions of ABA and cytokinins (CKs) on germination, stomata opening, photosynthesis, photorespiration, chloroplast development, and stress response are well documented (e.g. Khokhlova *et al.*, 1978; Kusnetsov *et al.*, 1994; Kulaeva *et al.*, 2002; Rivero *et al.*, 2009, 2010; Nishiyama *et al.*, 2011; Ha *et al.*, 2012).

ABA biosynthesis and function are closely associated with plastids. The first steps of ABA biosynthesis occur within plastids and generate xanthoxin, which is transported into the cytosol and oxidized to ABA aldehyde and finally to ABA (reviewed in Nambara and Marion-Poll, 2005). Low ABA levels coincide with an increased number of plastids per cell and higher amounts of lycopene in tomato fruits (Galpaz *et al.*, 2008). ABA is discussed as a factor involved in transmitting signals from the plastids to the nucleus (retrograde signalling; e.g. Hess *et al.*, 1997; Baier and Dietz, 2005; Chan *et al.*, 2010; Leister, 2012). ABA links environmental stress perception with the reduction of photosynthetic capacity (Seemann and Sharkey, 1987; Saibo *et al.*, 2009). Apart from restriction of CO_2_ availability by stimulation of stomatal closure as a short-term effect of enhanced ABA levels, long-term ABA effects on photosynthesis include the inhibition of thylakoid formation, chlorophyll biosynthesis, and Rubisco and PEP carboxylase activities (Lichtenthaler and Becker, 1970; Khokhlova *et al.*, 1978; Kusnetsov *et al.*, 1998).

ABA effects on photosynthesis correlate with reduced levels of corresponding nuclear transcripts. Studies made on the expression of specific nuclear genes coding for chloroplast proteins (e.g. Kusnetsov *et al.*, 1994; Staneloni *et al.*, 2008) are supported by results of genome-wide analyses demonstrating that ABA affects the activity of about 10% of *Arabidopsis* protein-encoding genes and that genes coding for chloroplast-localized proteins are enriched among the genes repressed by ABA (reviewed in Cutler *et al.*, 2010; Fujita *et al.*, 2011). Accordingly, nuclear gene-encoded proteins with function in photosynthesis and other processes within chloroplasts accumulate to altered levels following ABA treatment (Kusnetsov *et al.*, 1994; Rakwal and Komatsu, 2004; Wang *et al.*, 2010).

The major function of the chloroplast genome is to code for proteins involved in all steps of photosynthesis and the assembly of the photosynthetic apparatus (Bock, 2007). Considering the extensive studies on ABA-induced changes of nuclear gene expression and the role of ABA in stress-induced downregulation of photosynthesis, it is surprising that ABA and stress effects on the expression of chloroplast genes have not gained much attention so far (Saibo *et al.*, 2009). Solely, a preliminary study revealed inhibitory effects of ABA on the transcription of several etioplast genes in developing barley leaves (*Hordeum vulgare* L.) after short illumination (Kravtsov *et al.*, 2011).

This work analysed the influence of exogenous ABA on transcription and transcript accumulation in chloroplasts of basal and apical sections of young and mature barley leaves detached from light-grown seedlings. It demonstrates inhibitory effects of ABA on chloroplast gene expression at the levels of transcription and transcript accumulation. The repressive ABA effects were modulated by light and the developmental/metabolic state of the cells and plants. Furthermore, the CK 6-benzyladenine (BA) is shown to counteract the repressive effects of ABA by stimulating the transcription in chloroplasts.

## Materials and methods

### Plant material and hormone treatments

Barley seedlings (*H. vulgare* L. cv. Luch) were grown in a growth chamber in soil at 22 °C under a 16/8 light/dark cycle (white light of 130 μmol m^–2^ s^–1^) if not otherwise stated. The first leaves were detached from plants 4 or 9 d after sowing (4d-leaves and 9d-leaves). Detachment of leaves was performed for all experiments at the same time of the day to exclude effects of diurnal variations in RNA and hormone levels. ABA and BA (Sigma-Aldrich, St Louis, MO, USA) were dissolved in 96% ethanol; the final ethanol concentration in both control (water) and experimental variants was 0.096%. For incubation, the entire leaf blades floated on water or hormone solutions for 3 or 24h (Supplementary Fig. S1A, available at *JXB* online). In another set of experiments, primary 9d-leaves were preincubated on filter paper moistened with water for 24h and subsequently transferred to water or hormone solutions for 3h (Supplementary Fig. S1B). If not otherwise indicated, the detached leaves were kept under constant illumination (130 μmol m^–2^ s^–1^) during both pretreatment and treatment steps. To define a suitable ABA concentration, leaves were incubated for 24h with hormone concentrations ranging from 0.1 μM to 100 μM. Based on the results shown in Supplementary Fig. S2, ABA was applied at a concentration of 75 μM in further experiments (if not otherwise stated), which resulted in a distinct reduction of chloroplast transcriptional activity and is compatible with a previous study (Kravtsov *et al.*, 2011). BA was used at the concentration of 22 μM previously found to stimulate chloroplast transcription (Zubo *et al.*, 2008).

### Determination of chlorophyll content

The content of chlorophyll was determined according to Lichtenthaler and Wellburn (1983), separately in basal and apical segments after 0, 1, 2, and 3 d of incubation of the leaf blades on water or ABA solution.

### Chloroplast isolation, run-on transcription assay, and dot blot hybridization

**Fig. 1.**
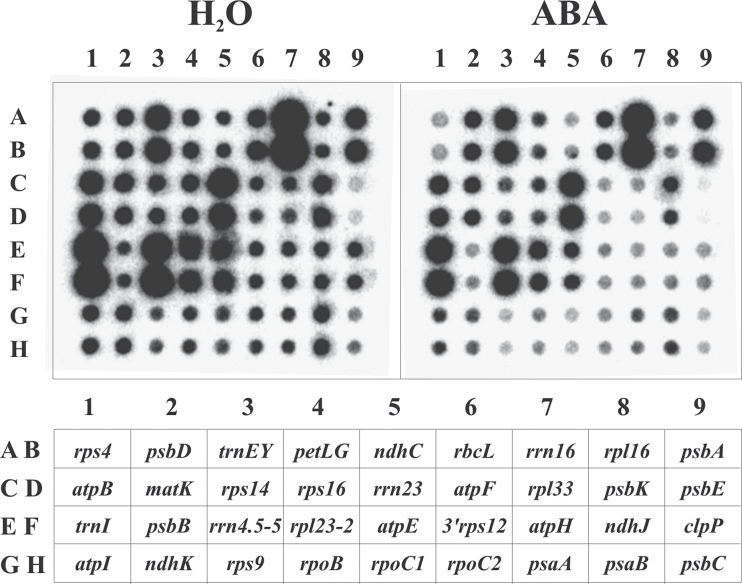
Effect of ABA on the transcription of chloroplast genes (run-on transcription assay). Leaves from 9-d-old plants were preincubated for 24h on water in light and subsequently incubated for 3h on water or ABA in light. Chloroplasts were isolated from basal sections of leaves and used for run-on transcription assays. ^32^P-labelled transcripts were isolated and hybridized to plastid gene probes (Supplementary Table S1) blotted onto nylon membranes according to the scheme shown at the bottom. All genes except *psbD* exhibited lower signals after treatment with ABA. The results of the quantification of hybridization signals are shown in Figs 2 and 3.

Chloroplasts were isolated from apical and basal sections (each 2cm in length) of the first leaves according to Zubo and Kusnetsov (2008). Run-on transcription assays were carried out as reported by Mullet and Klein (1987), modified as described previously (Zubo *et al.*, 2008). [^32^P]-labelled run-on transcripts were hybridized to 36 DNA sequences representing fragments of 40 chloroplast genes dot-blotted onto Hybond-N^+^ membranes (Amersham Pharmacia Biotech, UK) in two replicates (1 μg DNA per dot) using a Bio-Dot apparatus (Bio-Rad, USA) (Fig. 1). The selected chloroplast genes are listed in Supplementary Table S1 and shown in Fig. 1. For preparation of the gene fragments and more details, see Zubo *et al.* (2011a). A fragment of pUC57 was blotted to determine background hybridization signals.

### Isolation of RNA, RNA blot hybridization, and data quantification

Isolation of RNA, electrophoresis, blotting, and hybridization were performed as described (Zubo *et al.*, 2011b). The radioactive probe for the *HvS40* gene was prepared by PCR in the presence of [alpha-^32^P]-dCTP. The template was a gene fragment amplified by PCR from *HvS40* cDNA (Krupinska *et al.*, 2002). Radioactive probes for the chloroplast *rrn16*, *psbK*, *atpF*, *rps14*, *psaA*, and *psbD* genes were obtained by *in vitro* T7 transcription in the presence of [alpha-^32^P]-UTP using the MAXIscript T7 Kit (Ambion, Life Technologies, USA). The primers used to generate gene-specific PCR fragments (which served as templates for T7 transcription and PCR) are listed in Supplementary Table S2. All experiments were performed at least three times with independently prepared samples.

### Quantification of hybridization signals

Signals of the individual genes obtained by hybridization were quantified by scanning using the Molecular Imager FX with Quantity One software (Bio-Rad). The common logarithm of hormone/water ratios were calculated; the common logarithms +0.3 and –0.3 correspond to 2-fold up- and 2-fold downregulation, respectively. Total chloroplast transcription activity is the sum of the activities of the studied genes.

### Hormone extraction, purification, and determination

Phytohormones were extracted and purified according to Dobrev and Kaminek (2002). For analyses of endogenous CKs, 14-deuterium labelled standards were added (Apex Organics, Honiton, UK). A tritiated internal standard was used for the determination of ABA (Amersham, UK, specific activity 1.74 TBq/mmol, 5×10^3^ Bq).

Levels of ABA were determined using two-dimensional HPLC according to Dobrev *et al.* (2005). Quantification of ABA was performed by UV detection using a 235C diode array-detector (Perkin Elmer). HPLC-MS analysis of CKs was performed as described by Dobrev *et al.* (2002) using a TSQ Quantum Ultra AM triple-quad high-resolution mass spectrometer (Thermo Electron, San Jose, USA). Multilevel calibration graphs with [^2^H]-labelled CK internal standards were used for quantification. The detection limits of different CKs varied from 0.05 to 0.1 pmol/sample. Three independent experiments were done. Each sample was injected at least twice.

## Results

### ABA induces senescence in apical and basal segments of detached barley leaves

This study analysed basal and apical parts of 4d- and 9d-leaves. Detached barley leaves have been used for a long time as model system in research on plant hormones (e.g. Becker and Apel, 1993; Lee *et al.*, 1996) and have proved to respond sensitively to plant hormones with respect to chloroplast gene expression (Zubo *et al.*, 2008, 2011b; Zubo and Kusnetsov, 2008). Leaves of barley and of other grasses are particularly useful for analyses of age- and development-dependent effects of hormone action. They grow from a basal meristem and therefore exhibit a longitudinal age and developmental gradient with the youngest cells positioned at the leaf basis and the oldest cells in the apical zone. The basal leaf segments contain less chlorophyll than the apical ones (Supplementary Fig. S3) since their cells contain lesser plastids than the mesophyll cells in the apical parts. Moreover, the transitions from chlorophyll-free, photosynthetically inactive proplastids to mature, photosynthetically active chloroplasts and from heterotrophic (sink) to autotrophic (source) metabolism take place in the basal 2cm of barley leaves while mesophyll cells in the apical parts contain – as long as there is no senescence – exclusively photosynthetically active chloroplasts (Robertson and Laetsch, 1974; Baumgartner *et al.*, 1989).

In most experiments, detached leaves were incubated for 24h on water (control) or 75 μM ABA under continuous illumination (130 μmol m^–2^ s^–1^) (for the experimental design, see Supplementary Fig. S1). ABA has senescence-inducing and -accelerating activity (e.g. Aspinall *et al.*, 1967; Wingler and Roitsch, 2008; Zhang and Zhou, 2012). In order to find out if the conditions applied led to the expected responses, the effects of ABA on the chlorophyll content of leaves were assessed. The marked loss of chlorophyll in basal and apical parts of the ABA-treated leaves indicated the hormone-dependent triggering of senescence (Supplementary Fig. S3A). In the water control, only a weak loss of chlorophyll was observed in the oldest (apical) but not youngest (basal) parts of 9d-leaves (Supplementary Fig. S3A). ABA did not reduce the chlorophyll content in leaves from younger seedlings after 4 d of growth (not shown). Darkness is another inducer of senescence (Gan and Amasino, 1997). Incubation in darkness caused also a rapid drop in the chlorophyll content of leaves but had no additive effect to the ABA-induced chlorophyll degradation (Supplementary Fig. S3B). Senescence resulting from ABA treatment was further substantiated by the induction of the nuclear gene *HvS40* in the investigated leaf regions (Supplementary Fig. S4). HvS40 is a marker for senescence (Becker and Apel, 1993; Krupinska *et al.*, 2002) and has ABA-induced homologues in *Arabidopsis* (Fischer-Kilbienski *et al.*, 2010).

### ABA inhibits chloroplast transcription

Run-on assays measure the incorporation of labelled precursors into RNA during elongation (i.e. the transcriptional activity of genes; Mullet and Klein, 1987). Chloroplast RNAs were isolated and hybridized to 36 DNA fragments representing 40 chloroplast genes (Supplementary Table S1) dot-blotted onto nylon membranes (Fig. 1). The selected genes have functions in photosystem I (*psa* genes), photosystem II (*psb* genes), electron transport (*pet* genes), Calvin-Benson cycle (*rbcL*), ATP synthase (*atp* genes), NADH dehydrogenase (*ndh* genes), transcription (*rpo* genes), splicing (*matK*), translation (*rrn*, *trn*, *rpl*, *rps* genes), and protein degradation (*clpP*).

**Fig. 2.**
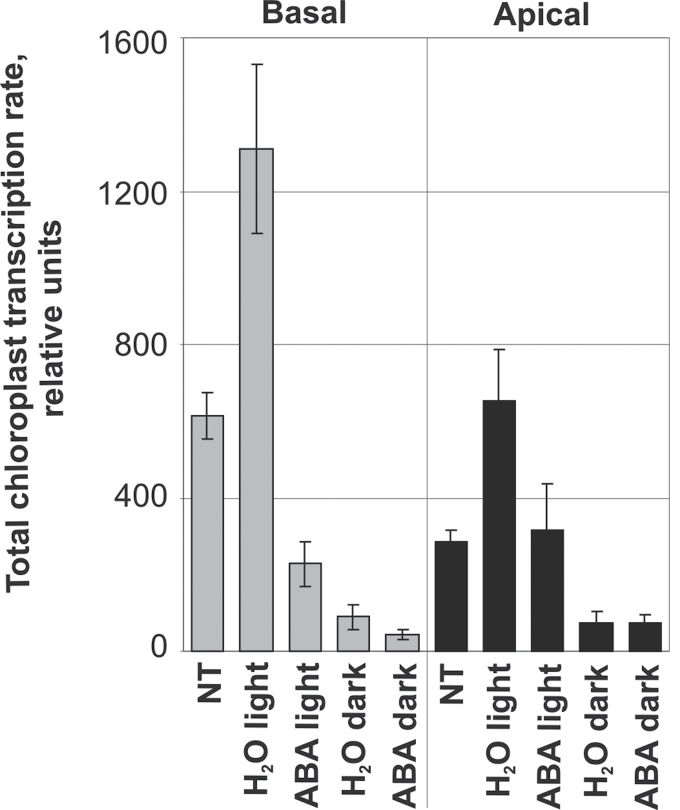
Run-on transcription of chloroplast genes: total transcriptional activities of chloroplast genes after 24h preincubation and short-term (3h) treatment with ABA (under light or in darkness). Isolated chloroplasts from basal and apical parts of the leaves were used in run-on transcription assays. Radioactive signals were detected and quantified as described in Materials and methods. Means of ABA/H_2_O ratios of transcription rates of all genes were calculated from the hybridization signals of three independent experiments for each condition.

**Fig. 3.**
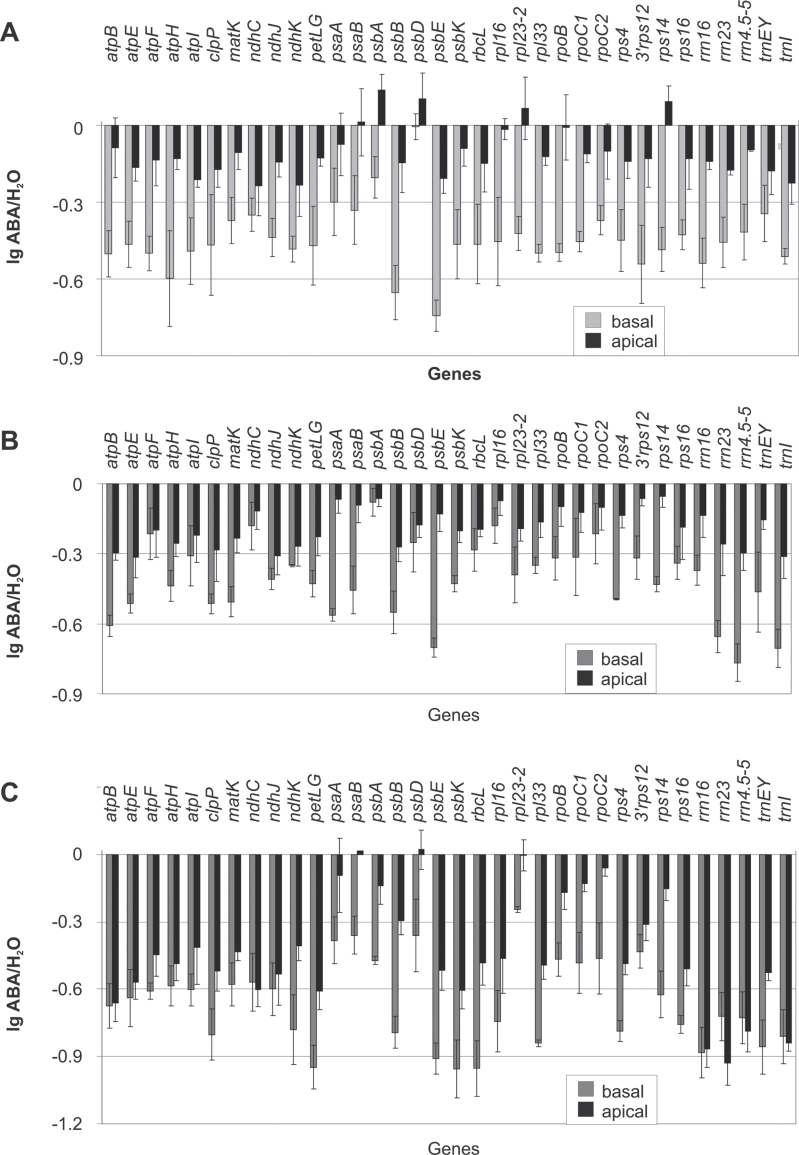
Run-on transcription of chloroplast genes: effects of ABA treatment in the light on the transcription of chloroplast genes in basal and apical sections of barley leaves. (A) Effects of short-term (3h) ABA treatment of leaves from 9-d-old plants after preincubation of the leaves on water for 24h under permanent illumination; original hybridization of one experiment is shown in Fig. 1. (B, C) Effects of long-term (24h) ABA treatment on leaves from 4-d-old (B) and 9-d-old plants (C). Common logarithms of the means with SDs are shown. All further experimental details as in Figs 1 and 2.

Preincubation of leaves for 24h on water and under continuous illumination (130 μmol m^–2^ s^–1^) resulted in about 2-fold activation of total chloroplast transcription and enhanced the sensitivity of chloroplast transcription to CK and methyl-jasmonate (Zubo *et al.*, 2011b). Therefore, the same experimental design (Supplementary Fig. S1B) was used to study potential effects of ABA on chloroplast transcription. If 9d-leaves were preincubated for 24h on water, quantification of the data revealed a strongly enhanced global transcriptional activity of chloroplasts in both basal and apical segments (Fig. 2), confirming the results of previous experiments (Zubo *et al.*, 2011b). The subsequent incubation for 3h on ABA under continuing illumination suppressed the transcription of nearly all chloroplast genes in the basal parts of the leaves (Fig. 1). The individual genes exhibited 2–5-fold reduction in transcriptional activity compared to the water control (Fig. 3A). A different result was obtained for the apical leaf sections. In untreated samples, the overall transcription activity of chloroplasts from the leaf apex reached only less than half of the activity observed in the basal parts (Fig. 2). The distinctly lower transcription rates in apical vs. basal sections were observed in all experiments and confirmed previous observations (Baumgartner *et al.*, 1989). In the leaf apex, ABA had no effect on the transcription of several chloroplast genes and repressed reproducibly the transcription less than 2-fold of the remaining genes (Fig. 3A). If not combined with preincubation for 24h on water, the short-term treatment with ABA for 3h alone had no significant effect on chloroplast transcription (data not shown). Thus, preincubation markedly increased the sensitivity of the leaves to ABA. The reason for this effect is unknown. It might be related to the activation of gene expression under this condition.

This work investigated whether a longer incubation with ABA could substitute for the preincubation. Long-term incubation with ABA (or water as control) was performed for 24h with 4d- and 9d-leaves. Repressive effects of ABA were observed in all samples (Fig. 3B, C). Like in the case of short-term (3h) treatment (Fig. 3A), the treatment for 24h had more drastic effects in the basal than the apical parts of leaves and in 9d- than 4d-leaves (Fig. 3B, C). ABA inhibited chloroplast transcription on average 2.6-fold in basal sections but only 1.5-fold in apical sections of the younger leaves (Fig. 3B), while the inhibition was 4.6-fold in the basal parts and 2.7-fold in the apical zone of the older leaves (Fig. 3C).

In general, ABA repressed housekeeping and photosynthesis genes to a similar extent. Nevertheless, this work also observed differences between individual genes with respect to their response to ABA. After short-term (3h) treatment, all investigated genes, except *psbD*, showed a markedly reduced transcriptional activity in basal segments (Fig. 3A). In the apical sections, *psbD* and also the photosynthesis genes *psaA*, *psaB*, and *psbA* and the housekeeping genes *rpl23/rpl2*, *rpoB*, and *rps4* exhibited no negative response after short-term and/or long-term incubation with ABA (Fig. 3).

### Light enhances the ABA effect on chloroplast transcription

Since ABA and light signalling interact (Chen *et al.*, 2008) and there is a strict dependence on light for the stimulation of transcription by CK (Zubo *et al.*, 2008), this work studied the potential requirement of light for ABA effects on chloroplast transcription. The total transcriptional activity of chloroplasts was markedly reduced during preincubation and incubation on water in darkness compared to the illuminated variant (Fig. 2) which is in agreement with the well-investigated light activation of several chloroplast genes while darkness decreases the association of the RNA polymerase with light-activated chloroplast genes (Yagi *et al.*, 2012, and references therein). The application of ABA in the dark did not significantly alter the transcription of chloroplast genes in the leaf apex (Fig. 2). In the basal segments, however, ABA reduced transcription even without illumination (Supplementary Fig. S5, Fig. 2), although to a lower extent than in illuminated leaves (Fig. 3A). The individual genes showed differential responses to ABA. While the activity of most genes was repressed more than 2-fold compared to the control, *psbD* did not respond significantly (Supplementary Fig. S5, black columns). After 24h long-term treatment with ABA, the average transcriptional activity of the chloroplast genes decreased about 2-fold compared to the water control (not shown; i.e. the effect was similar to the short-term treatment). Moreover, the lack of light during treatment modulated the pattern of ABA-repressed genes: the transcription of *psaA*, *psaB*, *psbA*, and *psbD* was more repressed while *petL/petG*, *rps4*, *rpoC1*, and *rpoC2* showed a lower repression compared to long-term incubation under illumination (Supplementary Fig. S5, grey columns; cf. Fig. 3C).

### ABA and cytokinins have antagonistic effects on chloroplast transcription

**Fig. 4.**
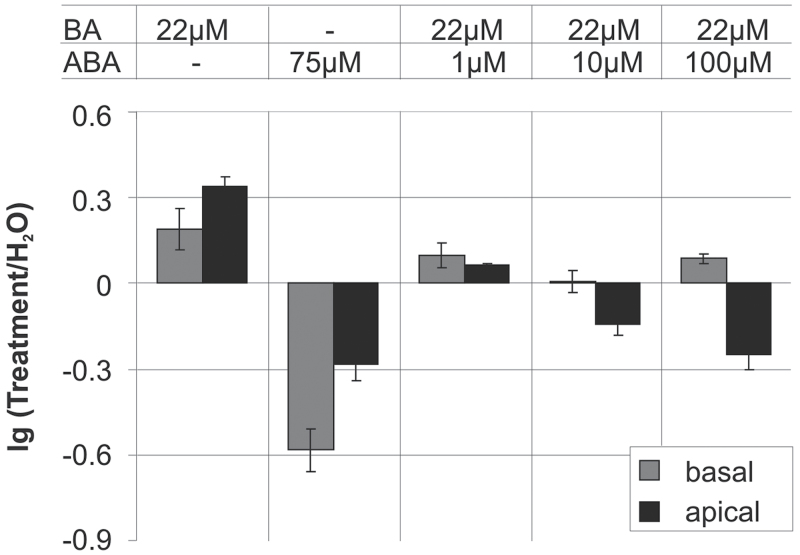
Run-on transcription of chloroplast genes in basal and apical leaf segments: effects of ABA and 6-benzyladenine (BA) and their combination on total chloroplast transcription activity in leaves from 9-d-old plants. Leaves were incubated for 24h on H_2_O, BA (22 μM), ABA (75 μM), or BA (22 μM) combined with different concentrations of ABA (1, 10, and 100 μM) in the light. Chloroplasts were isolated from basal and apical leaf segments. All further experimental details as in Figs 1 and 2.

The cytokinin BA has pronounced stimulating effects on the transcription of chloroplast genes (i.e. BA acts opposite to ABA; Zubo *et al.*, 2008). Therefore, this work checked for potential interactions between BA and ABA by treatment of 9d-leaves for 24h with combinations of BA and ABA. BA was applied at the concentration of 22 μM previously found to effectively enhance transcription and transcript accumulation in barley chloroplasts (Zubo *et al.*, 2008). ABA was added to the medium at concentrations of 1, 10, and 100 μM under continuous illumination (Fig. 4). In agreement with previous experiments (Zubo *et al.*, 2008), BA alone activated chloroplast transcription more strongly in apical sections while ABA inhibited transcription more effectively in basal segments. If applied simultaneously, the activating effects of BA were seen rather in the basal than the apical zone while the inhibitory action of ABA was more obvious in the leaf apex than in the basal parts. ABA counteracted the activating effects of BA in a concentration-dependent manner in the apical segments (Fig. 4). The highest ABA concentration investigated (100 μM) inhibited strongly the transcription in the apical segments, almost reaching the level of inhibition by ABA alone (Figs 3, 4) while it was not sufficient to inhibit completely the stimulatory effect of BA in the leaf base. Notably, the hormones showed a reversed action on the nuclear gene *HvS40*. BA was also found to suppress the ABA-induced expression of the senescence marker gene *HvS40* (Supplementary Fig. S4), which is in agreement with the well-investigated stimulation of senescence by ABA and its suppression by CKs.

### Hormone effects on steady-state levels of chloroplast RNAs

**Fig. 5.**
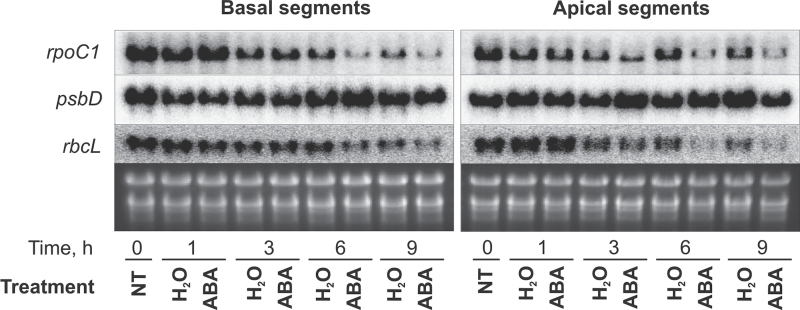
Effects of ABA on steady-state levels of chloroplast transcripts. RNA blot hybridization with probes for *rpoC1*, *psbD*, and *rbcL* was performed with samples from apical and basal segments of leaves from 9-d-old plants in the following variants: leaves preincubated for 24h on H_2_O in the light (H_2_O 24h) and treated after preincubation for further 1, 3, 6, or 9 h with H_2_O or ABA (75 μM) also in the light. Total RNAs were used as loading controls.

The regulation of RNA steady-state levels plays an important role in chloroplast gene expression. Steady-state levels of transcripts are not only controlled by the transcriptional activity of their genes but also by processing, stabilization, and degradation (Stern *et al.*, 2010; Barkan, 2011). To study whether the observed inhibition of the transcription rates by ABA resulted in comparable changes in the amount of chloroplast RNAs, steady-state levels were assessed by RNA blot hybridization. To allow for comparison with the data obtained for ABA effects on transcription rates after preincubation (Figs 2 and 3A), the time-course of ABA effects on transcript levels of three representative chloroplast genes, *rpoC1*, *psbD*, and *rbcL*, were studied in 9d-leaves after 24h preincubation on water under continuous illumination (Fig. 5). Preincubated leaves were incubated on 75 μM ABA for 1, 3, 6, or 9h under continuing illumination. Paralleling the situation observed for transcription rates, ABA reduced transcript amounts of *rpoC1* and *rbcL* mRNA strongly and the first effects were detected after 3h; however, while ABA had no significant effects on transcription of *psbD* (Fig. 3A), it even increased *psbD* transcript levels in both apical and basal segments (Fig. 5).

The effect of long-term treatment with ABA on steady-state levels of transcripts in 9d-leaves were analysed by RNA blot hybridization with and without illumination and in combination with BA for seven chloroplast genes (*rpoC1*, *rps14*, *rps16*, *psbK*, *psbB*, *psbD*, and *rbcL*). The results are described in detail in Supplementary Fig. S6. Compared to the water control, ABA suppressed the increase of mRNA levels of most studied genes in basal and apical leaf segments, whereas the *psbD* transcripts accumulated even to higher levels after ABA treatment. When the leaves were treated simultaneously for 24h with ABA and BA in the light (lanes ABA + BA), the transcript levels of *rpoC1*, *rps16*, *rps14*, and *rbcL* were lower than observed after treatment with BA alone, but higher than after application of only ABA. As previously observed for chloroplast RNAs in barley (e.g. Krause *et al.*, 1998), the transcript levels of the studied genes responded differentially to darkness (Supplementary Fig. S6). In comparison to the corresponding illuminated samples, the mRNA levels of *rpoC1*, *rps14*, *psbK*, *psbB*, and *rbcL* were lower both in basal and apical leaf sections after 24h incubation on water in the dark. ABA application in the dark further decreased the *rpoC1*, *rps14*, *psbK*, and *rbcL* mRNA levels. In contrast, the *psbB* and *psbD* mRNA levels increased in the dark and even more after ABA treatment. Taken together, the data on steady-state mRNA levels revealed that ABA and BA affect chloroplast transcript accumulation strongly and differentially. They show, moreover, that the hormone effects are modulated by the light conditions and the developmental state. While ABA clearly had more influence on transcription in the basal segments, the hormone affected RNA steady-state levels strongly also in the leaf apex (Figs 3 and 5 and Supplementary Figs S5 and S6).

### ABA alters the CK and ABA content in basal and apical leaf sectors

**Fig. 6.**
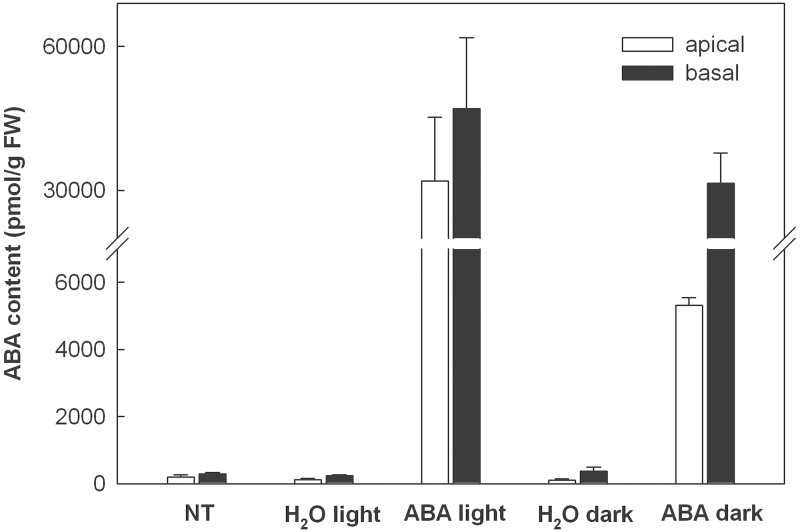
Effects of long-term ABA treatment on the content of ABA in basal and apical parts of leaves from 9-d-old plants grown in the light or dark. Detached leaves were incubated for 24h in the light or in the dark on water or ABA (75 μM). Freshly detached, not treated leaves served as control (NT).

Treatment with ABA may have influenced not only the endogenous level of this hormone but also the levels of CKs and other phytohormones. Vice versa, the endogenous hormone levels may have interfered with the action of exogenously provided ABA. Therefore, the contents of ABA and CKs were measured in basal and apical sections of 9d-leaves immediately after detachment (i.e. not treated, NT; Fig. 6) and after their incubation for 24h on water or 75 μM ABA in the light or in darkness. Incubation of leaves on ABA increased its endogenous content strongly, more in the basal than in the apical sections. In the dark, a pronounced lower accumulation of ABA was observed in the apical segments (Fig. 6 and Supplementary Table S3), probably due to its enhanced metabolism or reduced transpiration rate (Nilson and Assmann, 2007).

**Fig. 7.**
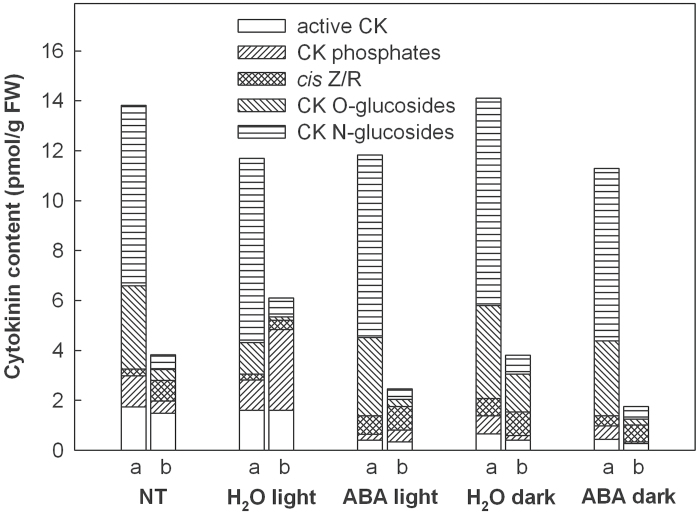
Effects of long-term ABA treatment on the content of different forms of CKs in apical (a) and basal (b) parts of leaves from 9-d-old plants grown in the light or dark. Detached leaves were incubated for 24h in the light or in the dark on water or ABA (75 μM). Freshly harvested, not treated leaves served as control (NT). The contents of CK-N-glucosides, CK-O-glucosides, *cis*-zeatin and its riboside, CK-phosphates, and active CKs were determined separately in apical and basal segments of the leaves by HPLC-MS (see Supplementary Table S3).

Relatively high levels of active CKs (*trans*-zeatin, isopentenyladenine, dihydrozeatin, and corresponding ribosides) were detected in both apical and basal sections of NT samples. The contents of *cis*-zeatin and its riboside were higher in the basal segments (Supplementary Table S3 and Fig. 7). Inactive CK metabolites, CK-N-glucosides (i.e. products of CK deactivation: *trans*-zeatin-7-glucoside, *trans*-zeatin-9-glucoside, dihydrozeatin-7-glucoside, and isopentenyladenine-9-glucoside), CK O-glucosides (i.e. storage forms: *trans*-zeatin-O-glucoside, *trans*-zeatin riboside-O-glucoside, dihydrozeatin-O-glucoside, and dihydrozeatin riboside-O-glucoside), and especially the by-far most abundant *cis*-zeatin (riboside) O-glucosides exhibited very high levels in apical segments. Incubation for 24h on water caused a significant drop of the level of *trans*-zeatin, the physiologically most active CK, in apical segments. *Trans*-zeatin was replaced by the less active dihydrozeatin (data not shown). ABA treatment for 24h in the light led to a strong decrease in the level of active CKs, including *trans*-zeatin, in both types of leaf segments. After 24-h incubation on water in the dark, the contents of active CKs decreased strongly, but to a lower extent than after ABA treatment in the light. Compared to the illuminated variant, ABA treatment in the dark did not lower the content of active CKs significantly (Fig. 7): i.e. similar to the situation described above for the chlorophyll content, no additive effect was observed for ABA and darkness with respect to the reduction of active CKs (for more details, see Supplementary Table S3).

## Discussion

### The expression of most chloroplast genes is repressed by ABA

These experiments revealed a remarkable sensitivity of chloroplast transcription to ABA (Supplementary Fig. S2). Concentrations in the range of 10 to 100 μM are commonly applied to elicit ABA effects on the activity of nuclear genes (e.g. Becker and Apel, 1993; Lin *et al.*, 2003; Fischer-Kilbienski *et al.*, 2010; Garg *et al.*, 2012). The treatment of rice seedlings with 100 μM ABA for 3h, for example, resulted in altered transcript levels of more than 3600 nuclear genes (Garg *et al.*, 2012). The current study group used a concentration of 75 μM ABA in most experiments to allow for comparison with a previous investigation of ABA effects on etioplasts (Kravtsov *et al.*, 2011). Interestingly, the transcription of mitochondrial genes was not affected under these conditions (M. V. Yamburenko, Y.O. Zubo, T. Börner, unpublished data). The response of the chloroplast genes to short-term (3h) ABA treatment was weak in the apex (Fig. 3A) but strong and very similar to the results of long-term treatment (24h) in the basal zone of 9d-leaves (Fig. 3C) indicating that a treatment for 3h is sufficient to downregulate the transcriptional activity of chloroplasts. Nevertheless, leaves were treated with ABA in most experiments for 24h to trigger a strong reaction to ABA in both the basal and apical sections. Kravtsov *et al.* (2011) reported recently that ABA inhibits the transcription of a few genes in barley etioplasts. The present experiments extend these findings by demonstrating that the transcription of genes is highly sensitive to ABA also in young and mature chloroplasts. Moreover, nearly all investigated genes responded with altered transcriptional activities and transcript levels to the hormone treatment.

The transcriptional machinery of plastids in higher plants is remarkably complex. In barley plastids, a *n*uclear gene-*e*ncoded phage-type plastid RNA *p*olymerases (NEP) and a *p*lastid gene-*e*ncoded bacteria-type RNA *p*olymerase (PEP) participate in the transcription of the plastid genome (Emanuel *et al.*, 2004). The PEP core subunits are encoded by the chloroplast *rpoA*, *rpoB*, *rpoC1*, and *rpoC2* genes. *rpoB*, *rpoC1*, and *rpoC2* together form an operon. While most chloroplast genes/operons possess PEP and NEP promoters and are transcribed mainly by PEP in photosynthetically active chloroplasts, *rpoB* is exclusively transcribed by NEP in barley chloroplasts (Zhelyazkova *et al.*, 2012). It is therefore particularly noteworthy that the *rpoB*, *rpoC1*, and *rpoC2* genes responded to ABA in all experiments by reducing their activity as most other investigated genes (Figs 1–3) indicating that ABA represses transcription by both PEP and NEP.

Although chloroplast gene expression is to a large extent regulated at the posttranscriptional level (Stern *et al.*, 2010; Barkan, 2011), there is also good evidence for transcription as target of regulatory processes (Lerbs-Mache, 2011; Liere *et al.*, 2011). In accordance with previous investigations on barley leaves (Rapp *et al.*, 1992; Baumgartner *et al.*, 1993; Krause *et al.*, 1998; Zubo *et al.*, 2008, 2011b), the current work revealed a good correlation between transcription rate and RNA accumulation (Figs 2–5, Supplementary Figs S5, S6) supporting the idea that transcriptional activity is an important determinant of RNA levels during leaf and chloroplast development. As previously shown for CK (Zubo *et al.*, 2008), there were, however, also clear differences between transcription and transcript accumulation with regard to the specific response of certain genes (e.g. *psbB*, *rps16*) and to the general weak response of transcription to ABA in apical segments under darkness contrasting the strong response of transcript levels under the same conditions (Figs 3, 5, Supplementary Figs S5, S6). Therefore, it is concluded that the phytohormones affect transcript levels not only via alteration of transcriptional activities but also independently of their action on transcription rates.

### Chloroplasts genes respond differentially to ABA

Transcription and transcript levels of the investigated chloroplast genes exhibited a differential response to ABA as was previously reported for other phytohormones (Zubo and Kusnetsov, 2008; Zubo *et al.*, 2011b). An originally high transcriptional activity is not a precondition for strong inhibition by ABA, as can be deduced from the reaction of the *psbE* gene that is only weakly transcribed but belongs together with the very actively transcribed *rrn* operon to the most strongly inhibited genes in basal tissues (Fig. 3). The majority of genes showed a stronger reduction of their activity in the basal vs. apical zone after long-term treatment (24h). Transcription of *trnI*, *atp*, *ndhC*, *ndhJ*, and the *rrn* genes, however, was reduced to a similar extent in the basal and apical segments of 9d-leaves (i.e. the developmental/metabolic state of the leaf section did not influence the response of these genes to ABA; Fig. 3C).

In either leaf part and at both investigated plant ages, the strongest inhibition of transcription was found for *rrn* genes and the *trnI* gene. These genes belong to the same operon. However, genes of the same operon may also respond differently to ABA (e.g. *psbD* and *psbK* in 9d-leaves; Fig. 3C). While *psbK* behaved like most other genes and was strongly inhibited, *psbD* belonged like *psbA* to the weakly repressed genes in basal sections and was not affected at all in the apex. This might be due to an ABA-dependent operon-internal promoter upstream of *psbD*. There are numerous operon-internal promoters in the barley chloroplast genome (Zhelyazkova *et al.*, 2012), and *psbD* has a specific, well-investigated blue-light and stress-activated promoter (Nagashima *et al.*, 2004). The *psbD* mRNA levels showed a deviating response, too. Whereas most genes revealed reduced transcript levels after ABA treatment, *psbD* and *psbB* transcripts even increased to higher levels after both long- and short-term treatment (Fig. 5 and Supplementary Fig. S6). There are several reports on expression patterns of *psbD* and *psbA* deviating from those of other genes. This might be related to their function in encoding proteins of the photosystem II reaction centre that undergoes continuous repair of light-caused damages (e.g. Krause *et al.*, 1998; Zubo *et al.*, 2008, 2011b; Mishev *et al.*, 2011; Mulo *et al.*, 2012). The differential response of chloroplast genes to ABA suggests that the ABA effect is not due to a general inhibition of RNA polymerase activity; rather it may be mediated via transcription factors and, in the case of transcript levels, of (de)stabilizing RNA binding proteins.

### Cytokinin, developmental state, and light affect ABA action on chloroplast gene expression

In agreement with previous results (Zubo *et al.*, 2008), treatment with BA had a stimulating effect on transcription and enhanced transcript levels in barley chloroplasts. This work extended the previous study by analysing the effects of simultaneous application of ABA and BA and obtained clear indications for counteracting activities of both hormones on transcription (Fig. 4) and RNA steady-state levels (Fig. 5 and Supplementary Fig. S6) in chloroplasts. The applied ABA concentration of 75 μM induced the expression of the nuclear *HvS40* gene strongly, an effect that was completely suppressed by the simultaneous treatment with 22 μM BA (Supplementary Fig. S4). The induction of *HvS40* is an indication of senescence (Becker and Apel, 1993; Krupinska *et al.*, 2002) and, together with the ABA-triggered loss of chlorophyll (Supplementary Fig. S3), demonstrates that the current conditions of foliar treatment with the hormone led to responses that could be anticipated from the well-investigated senescence-stimulating activity of ABA (e.g. Aspinall *et al.*, 1967; Fischer-Kilbienski *et al.*, 2010). Thus, the observed effects of ABA on chloroplast gene expression may well be part of the ABA-induced programming of metabolism and development in the direction of senescence. In agreement with this suggestion, this work observed a stronger inhibition of transcription by ABA in chloroplasts isolated from 9d- vs. 4d-leaves (Fig. 2 and data not shown). However, there might be also senescence-independent effects of ABA, as suggested by the much stronger repression of transcription in chloroplasts of basal (youngest cells) vs. apical (oldest cells) leaf sections (Figs 2 and 3 and Supplementary Fig. S2).

The different response of chloroplast gene expression in the basal and apical segments and under different light conditions to exogenously applied ABA and BA might be influenced by the state of the expression machinery itself, which is more active in the young parts of barley leaves (this report; Baumgartner *et al.*, 1989), activated by light and repressed in the dark (Fig. 2; Liere *et al.*, 2011). It might also depend on the state of hormone receptors and components of the hormone-triggered signalling chains (which has also not been investigated yet in different leaf regions) and/or on the endogenous levels of the hormones. This work therefore determined the levels of ABA and CKs in the same leaf material that was also used for the analysis of gene expression. As could be expected, application of ABA drastically increased the endogenous levels of this hormone (Fig. 6, Supplementary Table S3). The increase was more pronounced in the light than in the dark. ABA accumulation was particularly low in the apex after treatment in the absence of light (Fig. 6). These relatively low levels could explain the repression of chloroplast transcription by ABA under illumination in both basal and apical segments (Figs 2, 3) whereas in darkness ABA showed a significant effect only in the basal region (Fig. 2). In contrast to transcriptional activity, transcript amounts were affected by ABA in apical chloroplasts when leaves were kept in the dark (Supplementary Fig. S6). One may speculate that it needs a higher endogenous concentration of ABA to trigger a response of transcription than to alter transcript levels. Remarkably, ABA treatment induced a striking decrease in the content of active CKs both in the apical older and the basal younger leaf parts (Fig. 7; Supplementary Table S3). Darkness also reduced the content of active CKs in the leaves. Since BA stimulates chloroplast gene expression (Fig. 4, Supplementary Fig. S6), the decrease in active CKs by ABA treatment should support the ABA-induced repression of chloroplast transcription and changes of RNA steady-state levels. Thus, differing endogenous concentrations of ABA and CKs might cause at least part of the differences in the response of chloroplast gene expression to exogenously applied ABA and BA between the two leaf regions and between light and darkness.

The inhibitory effects of ABA on nuclear genes coding for proteins functioning in photosynthesis and related processes were previously shown (Kusnetsov *et al.*, 1994; Staneloni *et al.*, 2008; Cutler *et al.*, 2010; Fujita *et al.*, 2011). The current results demonstrate that ABA inhibits also the activity of most chloroplast genes. Since chloroplast genes are crucial for the assembly and function of the photosynthesis apparatus, it is proposed that one of the functions of ABA is to coordinate the expression of photosynthesis genes in the nucleus and the chloroplasts in response to internal and environmental cues. In this respect, ABA resembles photosynthetic redox signals, which affect chloroplast transcription and trigger retrograde signalling to the transcriptional machinery in the nucleus (Pfannschmidt and Yang, 2012). Since the synthesis of the ABA precursor xanthoxin takes place in the plastids (Nambara and Marion-Poll, 2005) and, since ABA content and action are influenced by the developmental state of the plastids/chloroplasts (Kulaeva *et al.*, 2002; Baier and Dietz, 2005; Hricova *et al.*, 2006; Robles *et al.*, 2012; Lee *et al.*, 2012; this report), this phytohormone may be a major player in the network of plastid-to-nucleus signalling.

Whether ABA acts on gene expression in plastids and the nucleus via the same or different signal transduction chains remains to be investigated. Since out of all components of the transcriptional machinery in chloroplasts only the PEP core subunits are coded for by chloroplast genes, the most likely scenario will be that signals resulting from altered endogenous concentrations of ABA (e.g. caused by stress or developmental processes) are transduced first to the nucleus. Among other nuclear genes also genes encoding transcription factors and/or other components involved in the regulation of chloroplast transcription and chloroplast RNA levels will change their activity and their products will in turn repress the activity of chloroplast genes and lower the levels of their transcripts. This study group is currently investigating ABA effects in *Arabidopsis* to learn more about the way(s) ABA acts on chloroplast gene expression.

## Supplementary material

Supplementary data are available at *JXB* online.

Supplementary Table S1. List of chloroplast genes and primers used to amplify gene fragments for blots used in run-on experiments

Supplementary Table S2. List of primers used to generate radioactively labelled probes for RNA blot

Supplementary Table S3. Content of different derivatives of CKs and ABA in apical and basal segments of 9-d leaves and treated for 24h with H_2_O or ABA in the light or in the dark

Supplementary Fig. S1. Experimental design

Supplementary Fig. S2. Effects of different concentrations of ABA on chloroplast transcription in basal and apical segments of 9-d leaves in the light

Supplementary Fig. S3. ABA effects on chlorophyll content in detached barley leaves

Supplementary Fig. S4. Effect of ABA and BA on the steady-state mRNA level of *HvS40* in the apical and basal parts of leaves incubated in the light or in the dark

Supplementary Fig. S5. Effects of long- and short-term ABA treatments on transcription of chloroplast genes in the dark

Supplementary Fig. S6. Effects of short-term treatment with ABA on steady-state RNA levels in chloroplasts

Supplementary references

Supplementary Data
