# Supplementary material for: Abscisic acid represses the transcription of chloroplast genes
Source: J Exp Bot. 2013 Sep 28;64(14):4491–502. doi: 10.1093/jxb/ert258 (PMC3808324; doi:10.1093/jxb/ert258)
Supplement: Supplementary Data [file supp_64_14_4491__index.html]

Abscisic acid represses the transcription of chloroplast genes — Abscisic acid represses the transcription of chloroplast genes — Supplementary Data 

# Abscisic acid represses the transcription of chloroplast genes

## Supplementary Data

Data files

**Files in this Data Supplement:**

- Supplementary Data - Supplementary Data
