# Supplementary material for: Abscisic acid represses the transcription of chloroplast genes
Source: J Exp Bot. 2013 Sep 28;64(14):4491–502. doi: 10.1093/jxb/ert258 (PMC3808324; doi:10.1093/jxb/ert258)
Supplement: Supplementary Data [file supp_ert258_jexbot104141_file001.pdf]

ABA represses the transcription of chloroplast genes

Maria V. Yamburenko, Yan O. Zubo, Radomíra Vanková, Victor V. Kusnetsov, Olga N. Kulaeva, Thomas Börner

Supplementary data

Supplementary tables

Table S1. List of chloroplast genes and primers used to amplify gene fragments for blots used in run-on experiments.

Gene	Primer sequence (5‘ – 3‘)	Position of amplified fragment in chloroplast genome
atpB	AGAGTAGAGACACTGCCGAT<br>GGCTGTAACATAGTTG	53027-54008
atpE	CGTATTACCAAACCACGCCCCTAT<br>TTCGCTTCAACCAAATCTTTCGTA	52314-52568
atpF	ACCAATGCCGAATCGACGACCTAT<br>TTCGAGTTGCTCAATGGTCCCTCT	33909-34429
atpH	GCTGCTGCTTCTGTTATTGCTGCT<br>CGCTAGTGCCACAACTAGTCCATA	32674-32877
atpI	TTCGGAATCCACAAACCATCCCGA<br>ATGCCGCTGACGTGAGTAAAGCTA	31520-31789
clpP	TCAGTATCGCAGTGCTTGTC<br>TTGGCTAATCTCAGGAATGG	68287-68685
matK	CTCGGTTAATCATCCTAATC<br>AAGTATCCTATGAGGAGAGT	2606-3523
ndhC	TTTGGACATTTCTAATAATAGCAAGCC<br>CTTTTCGCCATGCATAAACTAAAC	50117-50431
ndhJ	CAGGGTTGGTTATCTAATTGGCTA<br>GTTTAAGGCGCGGATGATT	48876-49260
ndhK	GGTAACAATGAAAATGGCTCCGT<br>TGTCGATGGTGATTGATAGAGCAA	49438-49807
petL/petG	CCCAGCTCTATTTATTGGCTTGAAC<br>ACGCAGTCACGAATAATCCCGCTA	64137-64434
psaB	TCTCACTTTGGGCAATTAGC<br>TTCCATCGAACGTACTCCCC	38974-39458
psbC	CGTGCATTTGTATGGTCTGG<br>TCACGATCGATTCCCTTTTC	10951-11552
psbK	TTGAGAGTGCGAATACAAGG<br>GCAAAAATAGGGTTAGGTGG	7122-7707
rpl16	GCTTCGTATTGTCGAGATCC<br>GTGTTATTGCTCTTCGTCCT	78503-79640

rpl23/rpl2	GTCATAAGCGCCTATACCGT<br>CACTTGTCCGACTGTTGCTA	81968-83046, 135088-136166
rpl33	CAAGGGAAAAGATGTTAGAATCA<br>CATATGAAATAATATCGACCGGAAATC	65799-66127
rpoB	AATTCGGATTGGCTCTTGGTC<br>CAACAATGCATTTCTCGGAC	21057-21803
rpoC1	AGGCCCAGCACTAAAAAACCCAC<br>GAATTCTCTATAATAATTCG	23799-23998
rpoC2	TGTCGGACCCTCAAGGACAAATGA<br>TTGTGCTCGAAATGGTTGTGCTCG	26070-26470
rps4	CTGACAGAACGACAATTAC<br>GACTATACGGCCATTAACT	45782-45973
rps9	AGTGAAGCGGAAGAACCAGA<br>AACAAAGCAAAACCGACACC	
3'rps12	AAAGATCTCCCTCCAAGCCGTACA<br>TGGAGCAGGCTACCATGAGACAAA	89911-90315, 127819-128223
rps14	AGTTTGATTCAGAGGGAG<br>GGATCTTGTTGCACCCGG	36949-37239
rps16	ATGCTCTTGGCTCGACATAG<br>CCTTCTCTTCGAGATCGAAC	5196-5970
rrn23	TAGGTTAGCCGAAAGATGGTTATAGGTTT<br>CGAGACAGTGCCCAGATCGTTAC	97930-98553, 119581-120204
rrn4,5/rrn5	GAAATAGGATAAGGTAGCGGCGAGAC<br>CAGGACCTCCCCTACAGTATCGTC	99436-99862, 118272-118698
trnE/trnY	TTATGACTTAGTGCGGGATG<br>CATAACTAGTATGTCGTGAT	15401-16224
trnI	CTCTCAGCCACATGGATAGTTCAA<br>CTGAAAGGAAGGGAGGATTAGGAA	94549-95032, 123102-123585

The probes for *psaA* (1058pb), *psbA* (769bp), *psbB*(1160bp), *psbD* (989bp), *psbE* (194bp), *rbcL* (1690bp), *rrn16* (1400bp) genes representing cloned restriction fragments of chloroplast DNA were kindly provided by Reinhold G. Herrmann (Institute of Botany, University of Munich, Germany). *psbC* and *rps9* were excluded from the calculations presented in Figures 2 - 4 and S2 because they showed high deviation between repetitions of run-on experiments.

Table S2. List of primers used to generate radioactively labeled probes for RNA blot hybridization.

Primer name	Primer sequence(5’–3’)
HvS40-for	CACCTACCTTCGCTCGCTAT
HvS40-rev	TGAGCAAGCTACTCGCACAC
rbcL-for	GCTGCCGAATCTTCTACTGG
rbcL-rev	GAAAAAGATACCGCGAGCAC
Hv-rpoC1-for	CGGAACAACAATCTTGCCTATCT
Hv-rpoC1-rev	cagagatgcataatacgactcactatagggagaTGATGTAATGAAAGCG
Hv-rps16-for	GTCTATCGAATCGTTGCAATTGA
Hv-rps16-rev	cagagatgcataatacgactcactatagggagaGCCTTCCTTAAAATATC
Hv-rps14-for	GAAGAAGCGGCAGAAATTAGAACAG
Hv-rps14-rev	cagagatgcataatacgactcactatagggagaCGGATAGCCCAAAAT
Hv-psbB-for	CATATTGCTGCGGGTACATTG
Hv-psbB-rev	cagagatgcataatacgactcactatagggagaGCTAAACCATTGCTAAC
Hv-psbK-for	GAAATGCTTGTTATGCCTAATATACTTAGTT
Hv-psbK-rev	cagagatgcataatacgactcactatagggagaGTACTAAAGATTTCATCGA
Hv-psbD-for	TTCCTTGTGCTTATTTCGCTTTAGG
Hv-psbD-rev	cagagatgcataatacgactcactatagggagaGCCTAATTGACACCAA
Hv-rrn16-for	CACATGCAAGTCGAACGGG
Hv-rrn16-rev	cagagatgcataatacgactcactatagggagaCAGCTACTGATCATCG

Table S3. Content of different derivatives of cytokinins (CKs) and ABA (pmol/gFW) in apical and basal segments of barley leaves detached from 9-day-old plants and treated for 24 h with H_2O or ABA (75 µM) in the light or in the dark (means of 3 independent preparations with SD; see also Figures 6 and 7 in the main text).

			**Active CKs**	**CK phosphates**	**CK O-glucosides**	**CK N-glucosides**	**Total *cis*-zeatin derivatives**	***cis*-zeatin and its riboside**	**ABA**
**Apical**		**NT**	1.74 ± 0.79	1.24 ± 0.32	3.33 ± 1.76	7.22 ± 0.53	245.98 ± 22.55	0.28 ± 0.11	200.82± 65.97
	**Light**	**H_2O**	1.61 ± 0.76	1.20 ± 0.17	3.01 ± 1.57	7.39 ± 0.68	205.59 ± 34.41	0.24 ± 0.15	124.63 ± 38.53
		**ABA**	0.40 ± 0.21	0.23 ± 0.04	3.14 ± 0.49	7.31 ± 0.49	214.13 ± 31.55	0.75 ± 0.40	31 941.12 ± 13318.74
	**Dark**	**H_2O**	0.66 ± 0.54	0.73 ± 0.20	3.72 ± 0.82	8.30 ± 1.57	264.93 ± 29.13	0.68 ± 0.65	106.86 ± 38.73
		**ABA**	0.45 ± 0.20	0.53 ± 0.13	2.99 ± 0.52	6.91 ± 0.58	235.53 ± 25.57	0.40 ± 0.14	5 325.53 ± 226.18
**Basal**		**NT**	1.49 ± 0.06	0.49 ± 0.11	0.43 ± 0.23	0.59 ± 0.13	117.25 ± 12.23	0.83 ± 0.25	289.47 ± 42.34
	**Light**	**H_2O**	1.60 ± 0.47	3.24 ± 0.34	0.12 ± 0.18	0.76 ± 0.34	109.54 ± 5.04	0.38 ± 0.05	245.46 ± 26.65
		**ABA**	0.34 ± 0.08	0.47 ± 0.18	0.30 ± 0.17	0.42 ± 0.10	102.83 ± 8.98	0.93 ± 0.43	47 023.21 ± 14823.51
	**Dark**	**H_2O**	0.40 ± 0.21	0.19 ± 0.06	1.51 ± 2.63	0.76 ± 0.27	79.49 ± 50.41	0.93 ± 0.66	373.62 ± 123.22
		**ABA**	0.28 ± 0.10	0.32 ± 0.52	0.25 ± 0.07	0.49 ± 0.10	87.49 ± 10.15	0.67 ± 0.30	31 488.44 ± 6296.11

ABA treatment increased drastically the endogenous content of ABA though to much lesser extent in the dark compared to illuminated leaves (Table S3; Fig. 6). Basal sections contained generally much less CK-N-glucosides (products of CK deactivation), CK O-glucosides (storage forms) and cis-zeatin derivates but more cis-zeatin and its ribosides than the apical parts (Table S3, Fig. 7). NT samples contained relatively high levels of active CKs, similar in apical and basal sections (Fig. 7, Table S3). Extremely high levels of CK N-glucosides (products of CK deactivation) and CK O-glucosides (storage forms of CKs) were detected in apical leaf sections. Their content in basal parts was much lower indicating that CK deactivation was less active in these segments. This was the main difference in CK metabolite content between older apical, and younger basal leaf parts. The level of CK phosphates (which can be both CK biosynthetic precursors and deactivation products) was also higher in the apical compared to basal sections of detached leaves. Interestingly, the content of *cis*-zeatin and its riboside was higher in basal segments (Fig. 7). Total *cis*-zeatin derivatives (mainly *cis*-zeatin O-glucoside) represented the most predominant CKs and were more abundant in apical segments (Table S3). Incubation for 24 h on water caused a significant drop of the *trans*-zeatin level, the physiologically most active CK, in apical segments. *Trans*-zeatin was replaced by the less active dihydrozeatin (data

not shown). The content of CK phosphates remained the same in apical segments, while it was strongly elevated in the basal ones (Fig. 7), which might be caused by the light-induced CK biosynthesis. The content of CK O-glucosides was reduced in basal segments. The content of CK N-glucosides remained similar to the value in NT plants in both leaf segments. ABA treatment for 24 h in the light led to a strong decrease in the level of active CKs, including *trans*-zeatin, in both types of leaf segments. The level of CK phosphates also decreased as an ABA effect. The contents of CK O-glucosides and N-glucosides remained in apical leaf sections at the same high level as in NT leaves. ABA treatment elevated the levels of *cis*-zeatin and its riboside in both leaf sections. After 24-h leaf incubation on water in the dark, the contents of active CKs and CK phosphates strongly decreased, but to lower extent than after ABA treatment in the light. The contents of CK O-glucosides and N-glucosides in apical leaf sections remained at the same level as in NT plants. In basal leaf sections, darkness markedly elevated the CK O-glucoside level. The levels of *cis*-zeatin and its riboside also were elevated in both leaf parts, which resemble the situation after ABA treatment in the light. ABA treatment in the dark only slightly lowered the content of active CKs, especially in comparison with the ABA effect in the light, probably due to the already strongly reduced content under the inhibitory influence of darkness. The content of CK phosphates was lower in the basal sections that in the apical ones, but the ABA effects were weaker than in the light. A weak decrease in the levels of *cis*-zeatin and its riboside was observed in both leaf parts (Fig. 7).

Supplementary figures

Legends

Figure S1. Experiment design

Two types of treatments of detached barley leaves are shown schematically. (A) Barley leaves detached from 4- or 9-day-old plants were incubated with hormones (ABA, BA, their combination, or H_2O supplied with corresponding amount of ethanol) for short- (3 h) or long-term (24 h) treatment in the light or in the dark. (B) Barley leaves detached from 9-day-old plants were preincubated on H_2O for 24 h and subsequently treated with ABA or H_2O for 3 h (short-term) in the light or in the dark.

Figure S2. Effects of different concentrations of ABA on chloroplast transcription in basal and apical segments of 9-day-old barley leaves in the light.

First leaves were detached from 9-day-old plants and incubated for 24 h on 0.1, 1, 10 and 100 µM ABA or H_2O in the light. Chloroplasts were isolated from basal and apical parts of the leaves and used for run-on transcription assays. ^{32}P-labeled transcripts were isolated and hybridized to chloroplast gene probes blotted onto nylon membranes (see Fig. 1, main text). The radioactive signals were detected and quantified as described in "Material and Methods". The experiments were repeated at least three times. Means of ABA/H_2O ratios of transcription rates of all the genes were calculated. Common logarithms of the means with ±SD are shown.

Figure S3. ABA effects on chlorophyll content in detached barley leaves.

To check physiological consequences of the ABA treatment for the leaf material, we analysed ABA effects on the chlorophyll content. ABA is known to promote senescence and one of the processes associated with leaf senescence is chlorophyll degradation (Evans *et al.*, 2010). First leaves were detached from 9-day-old barley seedlings and incubated in the light (A) or in the dark (B) on filter paper moistened with water (white circles and squares) or 75 µM ABA (black circles and squares) for 3 days. Chlorophyll content in basal (squares) and apical parts (circles) of leaves was analysed spectrophotometrically after extraction with acetone from freshly detached leaves (0 days) and after 1, 2 and 3 days of incubation. The means from three independent experiments performed in three replicates ± SD are presented; FW – fresh weight.

In the light, incubation of leaves on water resulted only in a slight decrease in the chlorophyll content of the apical parts, while no significant change was observed in the basal ones. Under the same conditions, ABA application caused distinct chlorophyll degradation in both leaf sections. It was stronger in the apical zone, which had higher initial chlorophyll content (Fig. S3A).

Incubation of the leaves in darkness on water induced chlorophyll degradation. This effect was stronger in the apical compared to the basal segments and enforced by incubation with ABA (Fig. S3B). Thus, senescence (as indicated by degradation of chlorophyll) progressed faster in the older compared to younger leaf parts as a result of ABA treatment and light deprivation. The impact of ABA on chlorophyll content depends also on the age of the whole leaf since we did not observe significant effects of ABA and darkness on the chlorophyll content in basal and apical segments of the younger and still developing primary leaves of 4-day-old seedlings (data not shown)

Figure S4. Effect of ABA and BA on the steady-state mRNA level of the *HvS40* gene in the apical and basal parts of barley leaves incubated in the light or in the dark.

Expression of the *HvS40* gene was previously shown to be a marker of leaf senescence in barley (Krupinska *et al*., 2002). We therefore analysed the transcript accumulation of *HvS40* as indicator for ABA-induced senescence. First leaves were detached from 9-day-old barley plants and incubated for 24 h on water, ABA (75 µM), BA (22 µM) or a combination of both hormones in the light or and in darkness. Equal amounts of total RNA isolated separately from apical and basal parts of the leaves were electrophoretically separated on agarose-formaldehyde gels and hybridized with a ssDNA probe for *HvS40*. Total RNA served as a loading control (bottom panels).

ABA strongly enhanced the transcript to similar levels both in the basal and apical sections, an effect that was less pronounced in illuminated leaves compared to leaves kept in darkness. Since CKs are well-known for their retarding effects on senescence (*e.g.* Kende, 1971), we checked also the effect the cytokinin 6-benzyladenine (BA) on *HvS40* expression. BA treatment alone did not result in a detectable effect on *HvS40* transcription. However, if applied together with ABA, BA prevented entirely the *HvS40* transcript accumulation. In contrast, hormone action on chloroplast transcription differed distinctly more between basal and apical segments and the same concentration of BA could not fully suppress the inhibition of transcription by ABA in chloroplasts of the apical segments (see main text, Fig. 4).

Fig. S5. Effects of long- and short-term ABA treatments in the dark. Run-on transcription was performed with chloroplasts isolated from basal parts of leaves detached from 9-day-old barley plants. Leaves were incubated for 24 h on ABA or H_2O in the dark (gray bars) or preincubated on H_2O for 24 h in the dark and subsequently incubated on ABA or H_2O for 3 h also in the dark (black bars). All further experimental details as in the legend to Figure 2 (main text).

Fig. S6. Effects of ABA and BA on steady-state levels of chloroplast transcripts. RNA blot hybridisations with probes for the chloroplast genes *rpoC1*, *rps14*, *rps16*, *psbK*, *psbB*, *psbD* and *rbcL* were performed separately with samples from apical and basal segments of leaves detached from 9-day-old seedlings in the following variants: freshly detached leaves (not treated samples, NT); samples incubated for 24 h on H_2O, ABA (75 μM), BA (22 μM), or a combination of ABA (75 μM) and BA (22 μM) in the light; samples incubated for 24 h on H_2O or ABA (75 μM) in the dark.

Supplementary references

Evans IM, Rus AM, Belanger EM, Kimoto M, Brusslan JA (2010) Dismantling of *Arabidopsis thaliana* mesophyll cell chloroplasts during natural leaf senescence. *Plant Biology (Stuttgart)* **12**, 1-12

Kende H (1971) The cytokinins. *International Review of Cytology* **31**, 301-338.

Krupinska K, Haussühl K, Schäfer A, van der Kooij TA, Leckband G, Lörz H, Falk J. 2002. A novel nucleus-targeted protein is expressed in barley leaves during senescence and pathogen infection. *Plant Physiology* **130,** 1172-1180

Figure S1

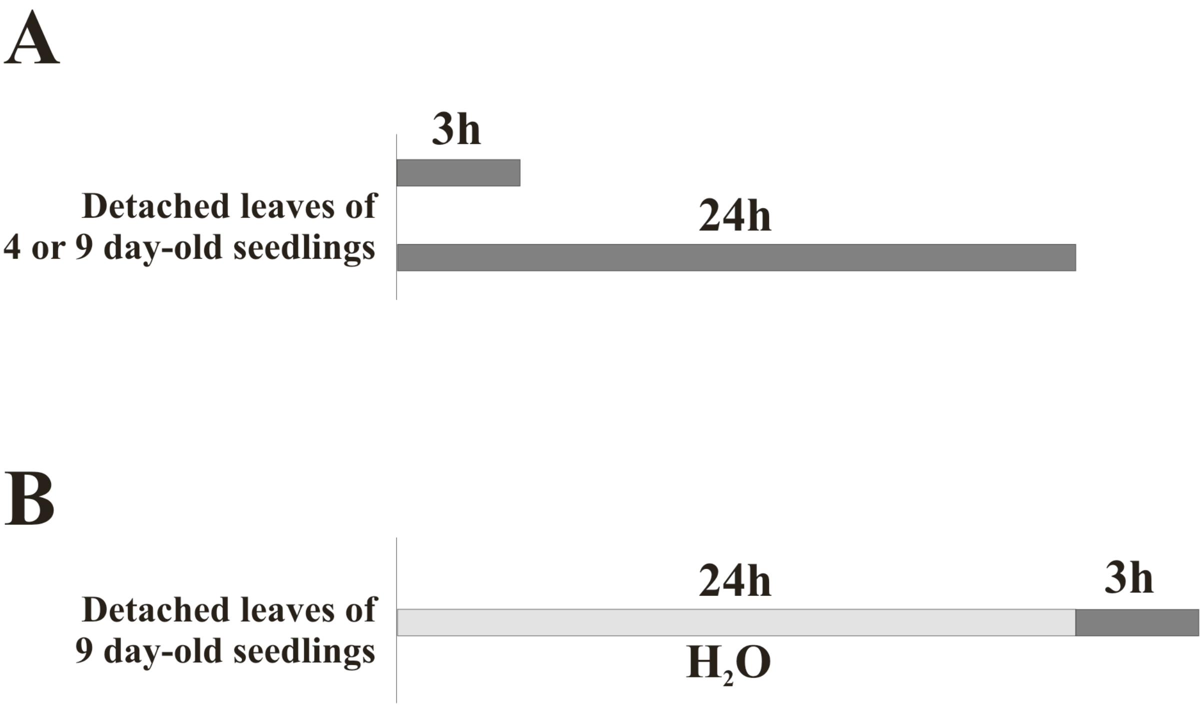
A
3h
Detached leaves of
4 or 9 day-old seedlings
24h
B
24h
3h
Detached leaves of
9 day-old seedlings
H2O

Figure S2

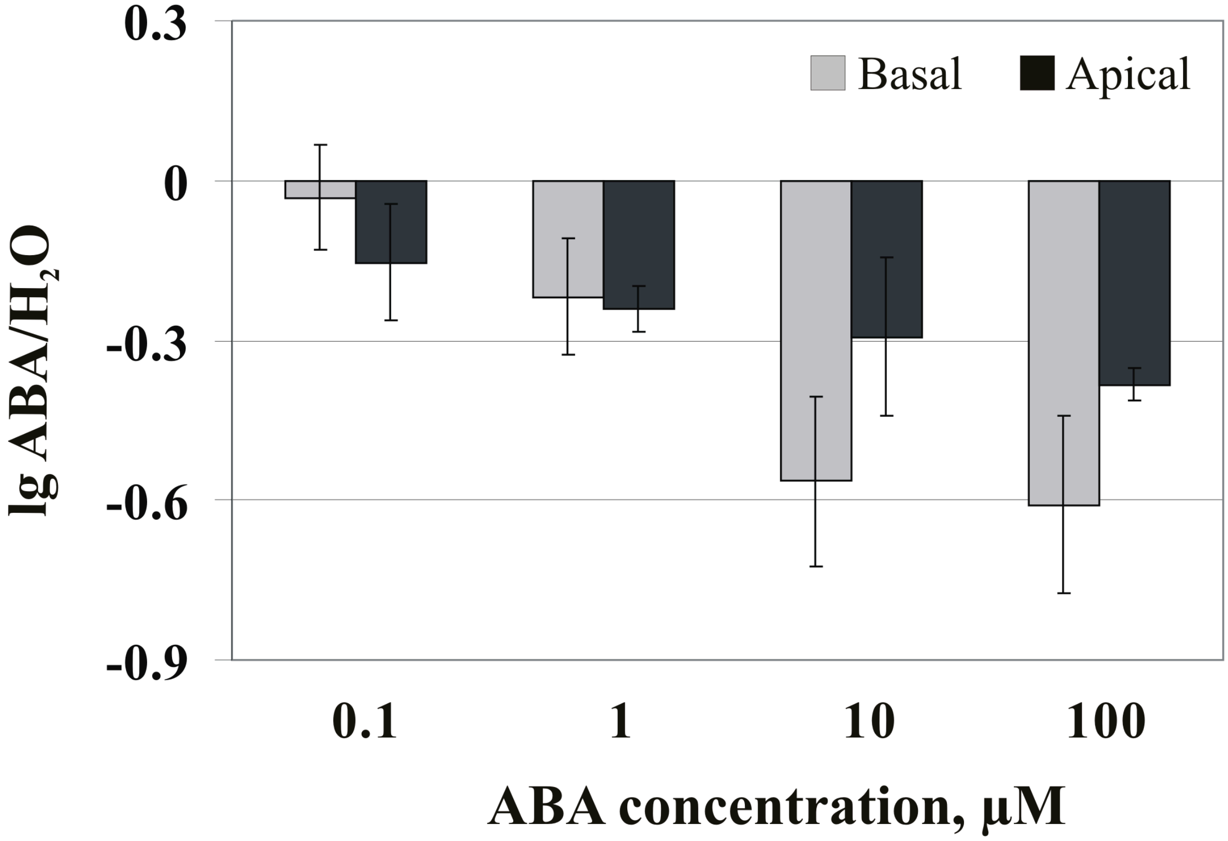

Basal
Apical
0.3
0
-0.3
-0.6
-0.9
lg ABA/H_2O
0.1
1
10
100
ABA concentration, μM

Figure S3

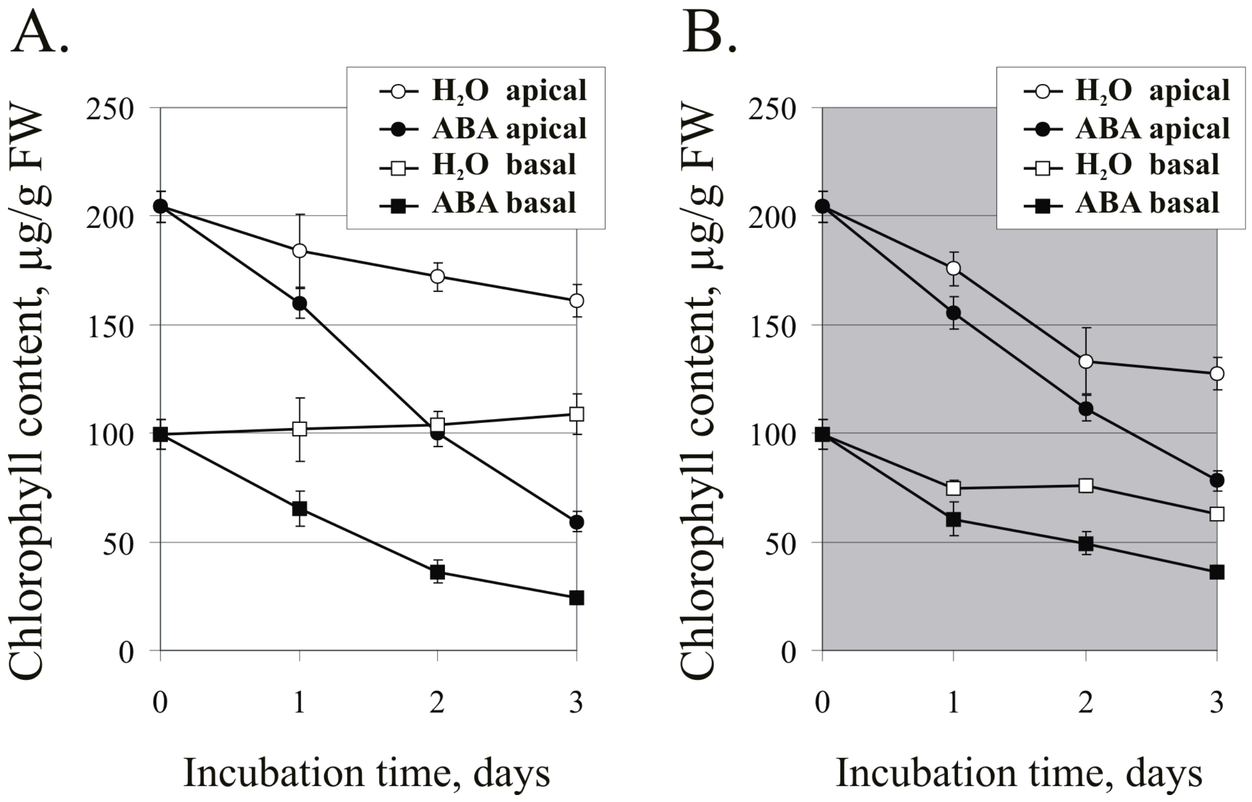
A.
B.
H2O apical
ABA apical
H2O basal
ABA basal
Chlorophyll content, µg/g FW
250
200
150
100
50
0
0
1
2
3
Incubation time, days

Figure S4

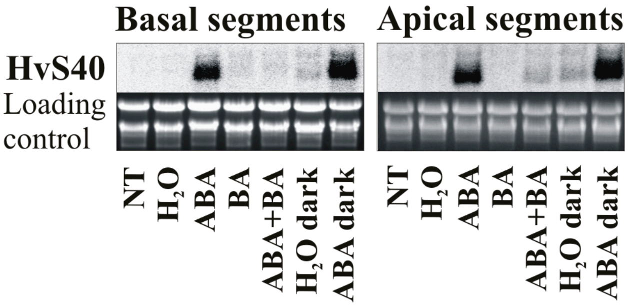

Basal segments
Apical segments
HvS40
Loading control
NT
H2O
ABA
BA
ABA+BA
H2O dark
ABA dark
NT
H2O
ABA
BA
ABA+BA
H2O dark
ABA dark

Figure S5

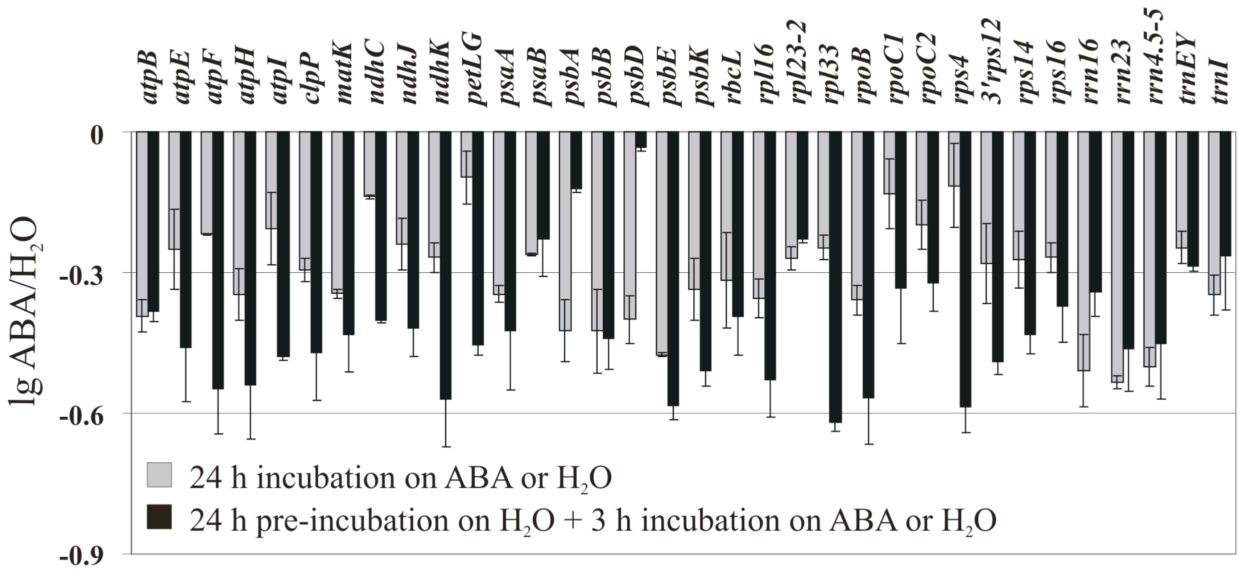
atpB
atpE
atpF
atpH
atpI
clpP
matK
ndhC
ndhJ
ndhK
petLG
psaA
psaB
psbA
psbB
psbD
psbE
psbK
rbcL
rpl16
rpl23-2
rpl33
rpoB
rpoC1
rpoC2
rps4
3'rps12
rps14
rps16
rrn16
rrn23
rrn4.5-5
trnEY
trnI
0
-0.3
-0.6
-0.9
lg ABA/H2O
24 h incubation on ABA or H2O
24 h pre-incubation on H2O + 3 h incubation on ABA or H2O

Figure S6

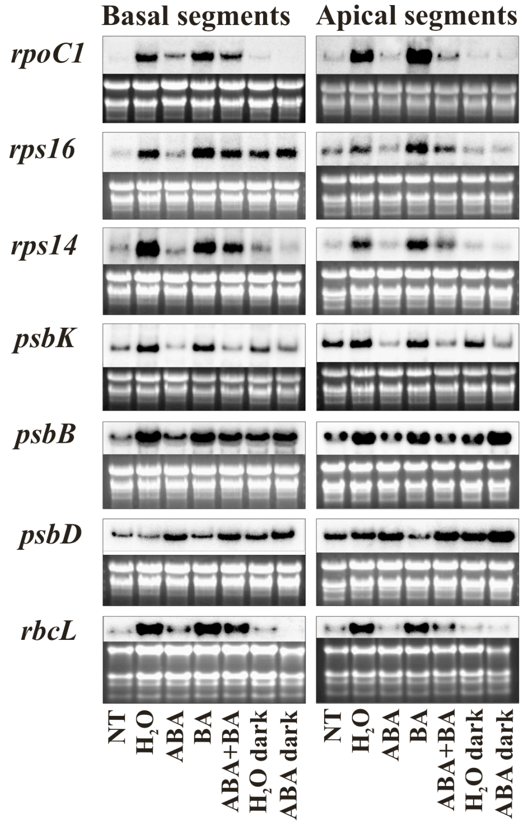

We assessed the steady-state levels by RNA blot hybridisation with probes for seven selected chloroplast genes (housekeeping genes: *rpoC1, rps14* and *rps16;* photosynthesis genes: *psbK*, *psbB, psbD*, and *rbcL*; Fig. S6). 9d-leaves were incubated for 24 h on water, ABA, and/or BA under continuous illumination or in darkness. The steady-state levels of the investigated RNAs responded differentially to the developmental stage of the tissues, to the hormones and the light conditions (Fig. S6). *Hormone treatment of illuminated leaves:* In comparison with freshly harvested leaves (lanes NT in Fig. S6) incubation for 24 h on water in the light (lanes H_2O) resulted in an increase of mRNA amounts of all studied genes, except *psbD*, in both leaf

segments (Fig. S6), corresponding to the enhanced transcriptional activities of the genes under these conditions (Fig. 2). Compared to the water control, ABA suppressed the increase of mRNA levels of most studied genes in basal and apical leaf segments (lanes ABA). ABA decreased the *psbK* mRNA level below the value of untreated leaves, whereas the *psbD* mRNA levels even increased after ABA treatment. BA increased mRNA levels of *rpoC1* and *rps14* in apical segments and of *rps16* in both segments (lanes BA compared with lanes H_2O). In contrast, BA reduced the *psbD* mRNA levels in the apical segments. BA did not affect markedly the other transcripts. The differential effects of BA on transcript steady-state levels confirm previous observations. When the leaves were treated simultaneously for 24 h with ABA and BA in the light (lanes ABA + BA), the transcript levels of *rpoC1*, *rps16*, *rps14*, and *rbcL* were lower in both leaf segments than observed after treatment with BA alone, but higher than after application of only ABA. The amounts of *psbK* and *psbD* transcripts were similar to the levels found after treatment with ABA alone, relatively low for *psbK* mRNA and relatively high for *psbD* mRNA. Simultaneous ABA and BA application had different effects on *psbB* mRNA in apical and basal leaf sections: BA partially compensated the inhibitory effect of ABA on transcript accumulation in the basal segments, while no obvious BA effect was seen in the apical segments, i.e., the *psbB* mRNA levels were as low as in the ABA treated variant.

Hormone treatment in darkness: The transcript levels of the studied genes responded differentially to darkness. Compared to freshly removed leaves (lanes NT in Fig. S6), 24 h of darkness did not change significantly the transcript levels of *rps14, rbcL, psbK,* reduced (*rps16* in apical segments) or raised them markedly (*rps16* in basal segments) or only slightly (all other; lanes H_2O dark). In comparison to the corresponding illuminated samples (lanes H_2O), the mRNA levels of *rpoC1*, *rps14*, *psbK*, *psbB,* and *rbcL* genes were lower both in basal and apical leaf sections after 24 h incubation on water in the dark. ABA application in the dark further decreased the *rpoC1*, *rps14, psbK,* and *rbcL* mRNA levels in basal segments and those of *psbK* and *rbcL* mRNAs also in the apex (lanes ABA dark). In basal sections, no substantial difference was observed between *rps16* mRNA levels after light and dark treatments. In apical segments, however, *rps16* mRNA levels were considerably lower when the leaves were kept in darkness vs. light. Interestingly, ABA application in the dark had no significant effect on the accumulation of *rps16* transcripts in both leaf sections. In contrast, the *psbB* and *psbD* mRNA levels increased in the dark and even more after ABA treatment.
